# Letter to the editor of: “posterior tibial plateau impaction fractures are not associated with increased knee instability: a quantitative pivot shift analysis”

**DOI:** 10.1007/s00167-023-07361-8

**Published:** 2023-03-22

**Authors:** M. Krause, A. Korthaus, J. Frings, M. T. Berninger, T. C. Drenck, L. Eggeling, R. Akoto, K. H. Frosch

**Affiliations:** 1 Department of Trauma and Orthopaedic Surgery, University Medical Center Hamburg-Eppendorf, Martinistraße 52, 20251 Hamburg, Germany; 2 Department of Trauma Surgery, Orthopaedics and Sports Traumatology, BG Hospital Hamburg, Hamburg, Germany

Dear editor,

With great interest we have read the recent publication of Godshaw et al. and their analysis of the association of the posterolateral tibial impression fracture (PLIF) and the pivot shift phenomenon [1]. As the authors highlight, this topic has come into recent focus due to an increased understanding of posterolateral tibial plateau fractures and their potential association with outcome of anterior cruciate ligament (ACL) ruptures. The authors included 284 patients that were subdivided into a fracture and a non-fracture group. The fracture group was defined as “articular-impaction” or “displaced-articular fragment” with a displacement of at least 2 mm. The authors found no statistical difference among the three groups with respect to ap translation or pivot shift acceleration [1].

From the “ligament” point of view, multiple biomechanical and clinical studies have highlighted the association of a steep lateral tibial slope on the risk for ACL rupture, including numerous publications of the same study group [2–6].

From the “bony” point of view, thanks to the CT-morphological assessment of posterior fracture runs, modern classifications such as the ten-segment-, revisited Schatzker or updated-three-column classification have not only led to a paradigm shift in the surgical strategy, but also in the quality of reconstruction, not limited to posterolateral tibial plateau fractures [7–12]. In addition, the presented findings stand in contrast to results of Flury et al. who showed that PLIF size matters with regard to ACL reconstruction outcome [13]. Hence, we would like to address major limitations of the study and question its scientific value for this important issue that has still to be answered with appropriate measures.

First, given the high risk of malreduction and its impaired knee function in the treatment of PLIF, their treatment has received lots of scientific attention [14, 15]. Several established classifications have outdated the classifications named by the authors [7, 10, 12]. There are numerous descriptions of posterolateral approaches resulting in specific concepts to address PLIF as needed [10, 16–20]. Hence, we strongly disagree with the authors’ introductory statement that “almost all [of PLIF] heal” and “surgical fixation of these fragments is usually not performed”.

Second, Bernholt et al. as well as Menzdorf et al. have proposed different classifications of PLIF with respect to combined ACL injuries [21, 22]. Both classifications acknowledge the width next to the depression of the posterolateral fragment (Fig. 1.). Hence, regarding theoretical concepts of PLIF and its influence on ACL outcome, size seems to matter. Both classifications include the hypothesis of submeniscal support and have found significantly more lateral meniscus lesions in larger depressions involving at least 50% of the submeniscal bony support [21, 23]. The negative impact of severe posterolateral meniscus lesions on the pivot shift phenomenon is biomechanically and clinically well established [24, 25]. There is also clinical evidence that severity of PLIF depression has an influence of the outcome of ACL reconstruction [13]. Intraarticular reconstruction in patients with ACL rupture and third-degree pivot shift showed excellent clinical outcome and no failure after 18.2 ± 13.5 months [22] (Fig. 2).

**Fig. 1:**
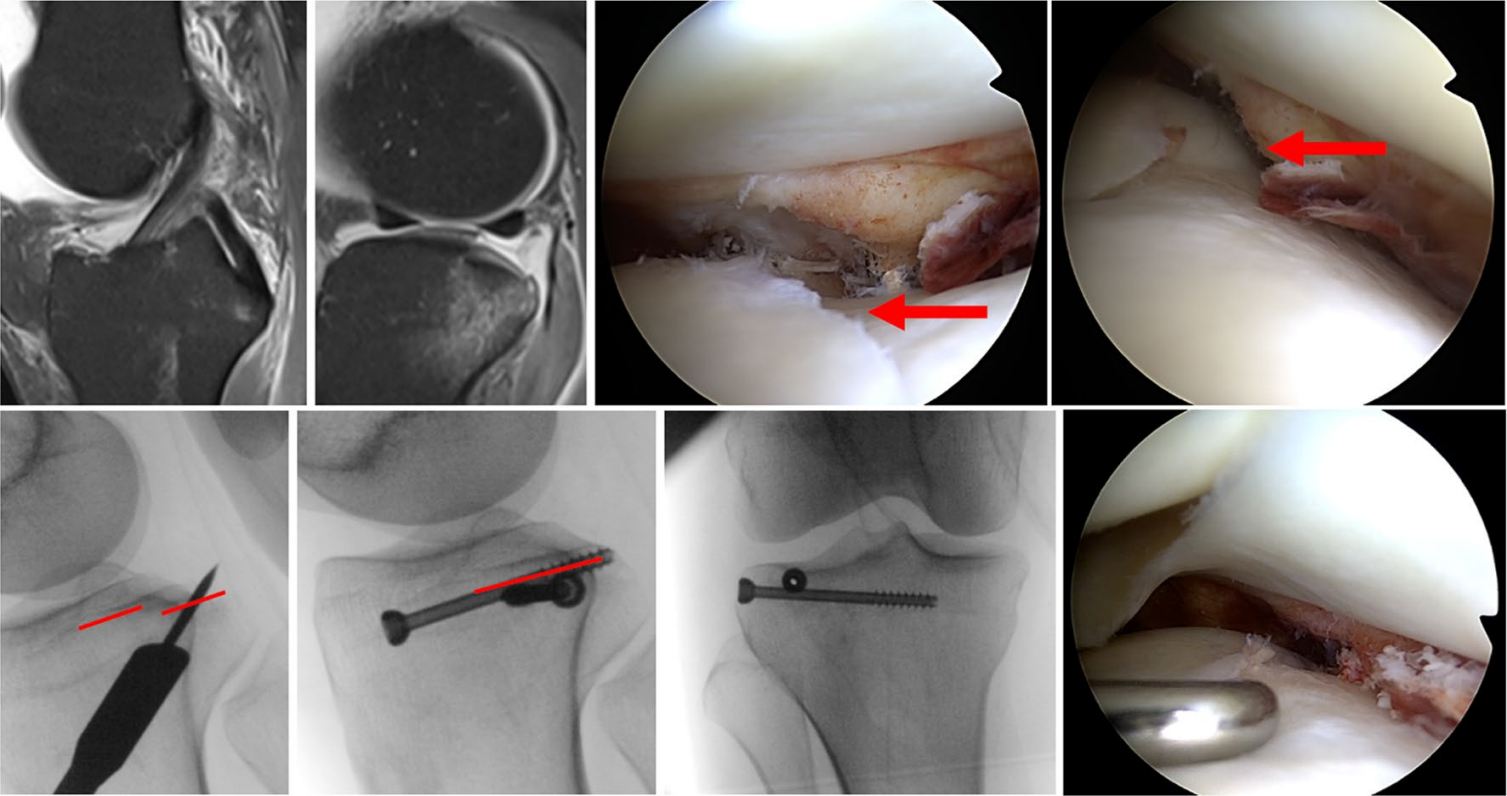
A 31-year-old female after skiing trauma with negative Lachman and intact ACL, but positive pivot shift due to isolated displaced PLIF type 2c [Menzdorf et al.[22]] or type IIIB [Bernholt et al. [21]]. MRI and arthroscopic findings (red arrows) show the PLIF of more than 2 mm depression and more importantly the complete width of the posterior horn of the lateral meniscus (upper row). Intraoperative findings demonstrate arthroscopic anatomic reduction and minimally invasive fixation as well as improved bony support of the posterior horn of the lateral meniscus (ARIF, lower row)

**Fig. 2:**
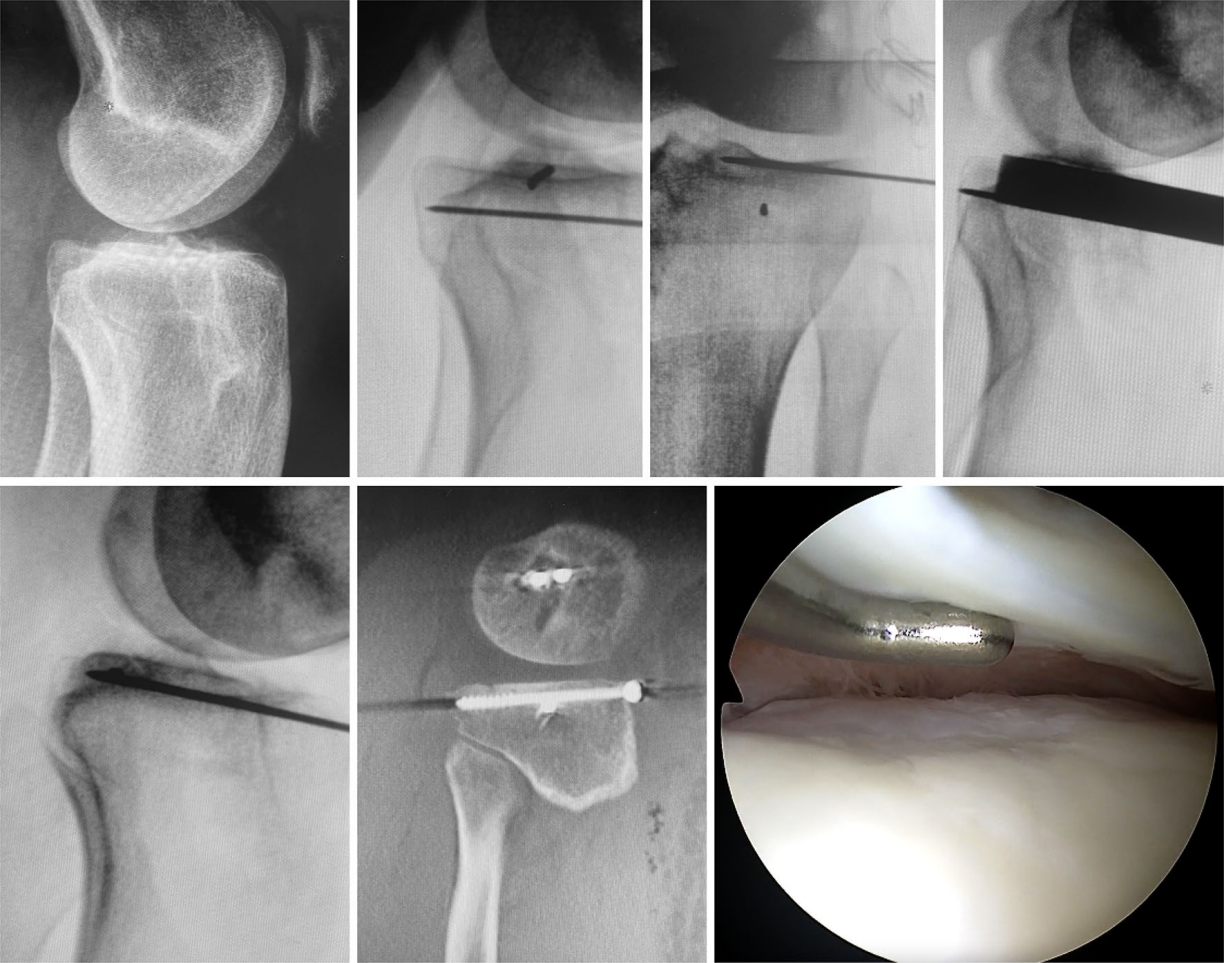
A 32-year-male with revision non-contact ACL rupture und PLIF type Bernholt IIb or Menzdorf type 1c. Two-stage surgery with intraarticular osteotomy of the posterolateral quadrant and tunnel filling, followed by revision ACL reconstruction with anterolateral tenodesis 4 months later. Arthroscopic follow-up at revision ACL reconstruction shows sufficient bony submeniscal support

Based on the theoretical and clinical background, it seems incomprehensible to us why the authors did not make a further distinction with respect to PLIF size and its relation to the posterior horn of the lateral meniscus. Looking at the figures illustrating either a “depressed” and “displaced” PLIF, both cases represent non-displaced fractures, which naturally do not have any impact on knee kinematics. In conclusion and unfortunately, this study may not have included patients with PLIF with a severity of at least Bernholt type III or Menzdorf type 1c, ≥ 2b, or 3b. Therefore, the authors were not able to answer their hypothesis on “how sufficiently [PLIF] contribute to rotatory knee laxity using quantitative pivot shift analysis”. Future studies should classify PLIF according to established classifications to test their potential association with the outcome of ACL reconstructions.

## Data Availability

As this is a comment on an original study, we have no data to present.
